# Staying physically active after spinal cord injury: a qualitative exploration of barriers and facilitators to exercise participation

**DOI:** 10.1186/1471-2458-9-168

**Published:** 2009-06-01

**Authors:** Matthew Kehn, Thilo Kroll

**Affiliations:** 1 National Rehabilitation Hospital (NRH), Research Division, Washington, DC, USA; 2 University of Dundee, Alliance for Self Care Research, School of Nursing & Midwifery, Dundee, UK

## Abstract

**Background:**

While enhancing physical activity has been an essential goal of public health officials, people with physical impairments such as spinal cord injury (SCI) are more likely to live a sedentary lifestyle. Exercise has been shown to decrease the risk for many of the secondary conditions associated with SCI, including osteoporosis, cardiovascular disease, pressure ulcers, urinary tract infections, diabetes and arthritis, yet this population is rarely a target for health promotion efforts. This paper examines the self-reported exercise experiences of people with SCI using a qualitative-exploratory design.

**Methods:**

We enrolled 26 individuals with SCI (15 self-described 'exercisers' and 11 'non-exercisers') from a non-random pool of survey responders. Semi-structured phone interviews were conducted to record participants' experiences with exercise pre/post injury, barriers and facilitators to being active and perceived health impact.

**Results:**

Regardless of exercise status, all participants reported physical activity prior to injury and expressed interest in becoming active or maintaining an active lifestyle. Participants identified a range of both motivational and socio-environmental factors that were either facilitating or constraining of such a lifestyle. Non-exercisers identified barriers to exercise, including a perceived low return on physical investment, lack of accessible facilities, unaffordable equipment, no personal assistance and fear of injury. Exercisers identified facilitators, including personal motivation, independence, availability of accessible facilities and personal assistants, fear of health complications, and weight management. Exercisers associated a greater range of specific health benefits with being active than non-exercisers.

**Conclusion:**

Despite motivation and interest in being exercise active, people with SCI face many obstacles. Removal of barriers coupled with promotion of facilitating factors, is vital for enhancing opportunities for physical activity and reducing the risk of costly secondary conditions in this population.

## Background

More than a decade ago, the United States National Institute of Health Consensus Conference on Physical Activity (1995) concluded that 'All Americans should engage in regular physical activity'[1]. Enhanced physical activity is often identified as a key public health objective and leading health indicator and yet research has shown that, upon returning to the community after rehabilitation, people with activity limitations, such as spinal cord injury (SCI), are less likely to be physically active when compared to the able-bodied population[2,3]. Trend data tracking physical activity among people with disabilities between 1997 and 2006 put forward by the U.S. Centers for Disease Control and Prevention show that Healthy People 2010 activity targets are largely missed as activity rates continue to stagnate[4]. Ranking at the lower end of the physical activity spectrum, people with SCI are at a heightened risk for ill health and costly secondary conditions [5-7]. As a group, people with disabilities rarely feature as a target for health promotion efforts[8].

Acute mortality rates for people with spinal cord injury (SCI) have declined over the past three decades[9] while clinical attention has increasingly focused on the prevention of secondary conditions. It has been established that people with SCI are highly susceptible to medical complications and secondary chronic conditions, such as pressure ulcers, urinary tract infections, diabetes, cardiovascular disease, obesity, osteoporosis and arthritis [10-16], and that physical activity can help prevent such conditions[17], enhance functional abilities [18-20] and increase quality of life and social integration [20-22].

The path towards a physically active lifestyle however, is fraught with obstacles for people with disability. Several barriers to exercise have been identified in prior studies, including accessibility, pain, costs, psychological barriers, a lack of motivation and energy, and a lack of logistical information [23-26]. Other determinants of physical activity identified include completeness and level of injury[2,27], intention and perceived behavioral control[28,29], and the presence of health complications[25]. Factors working against these barriers to facilitate an exercise active lifestyle have also been identified, including preparation during time in rehabilitation and the role of peer mentors[25,30]. An increase in quality of life, energy, self-confidence and body image were reported by individuals with SCI participating in a structured exercise training program[31] while health and fitness have been identified as reasons for continued participation in exercise[30].

While existing research on exercise after SCI provides valuable insight towards identifying determinants, much of this research has been done in a controlled setting, through a structured exercise program, or in relation to rehabilitation discharge. Studies have used a range of standardized means to measure physical activity, including a structured reporting scale[24,32], a validated self-report instrument[24,25,28], an activity monitor[2] or by providing exercise parameters to use in self-identification (e.g. 30 minutes per day, etc.)[27,29]. In contrast, this study asked participants to self-identify as 'exercisers' or 'non exercisers', thus taking into account the perceived identity of individuals as 'physically active' individuals rather than professionally defined categories. It may be assumed that self-definition of physical activity may broaden the range of barriers and facilitators that could be identified. Additionally, studies have primarily focused on perceived benefits and barriers, with less attention on perceived facilitators. It is unclear why some people continue to be physically active and pursue exercise opportunities after their injury while others do not. While exercise is essential to enhancing health, the onset of a physically disabling condition can provide serious challenges towards an active lifestyle. Knowledge of factors that may enhance exercise participation after SCI is crucial, particularly in order for health professionals to adequately tailor their support and interventions to the needs and lifestyle of their patients, thus improving upon the long-term rehabilitative process. For this reason, this paper reports on findings from a qualitative study aimed at identifying the factors that people with SCI living in the community perceive as affecting their level of physical activity. More specifically, the study sought to identify barriers to and facilitators of exercise as well as any perceived links between physical activity and secondary health conditions.

## Method

This study is based on a qualitative-exploratory design following a quantitative exploration of exercise and physical activity patterns.

### Sampling

Twenty-six adults with spinal cord injury were recruited (15 self-reported 'exercisers' and 11 'non-exercisers'). This sample size was deemed sufficient to explore the topical scope addressed in this paper, and is consistent with similar studies reported in the literature[33,34].

Participants were selected from a pool of 592 nation-wide survey participants dependent upon whether they self-identified as an 'exerciser' or 'non-exerciser'. The survey pool itself was non-random and consisted of self-selected participants who met the following criteria:

- 1 year post SCI

- 18+ years of age

- Speak English

- Consent to being interviewed

Researchers randomly contacted eligible participants by telephone or email to verify eligibility, willingness to participate, status as an 'exerciser' or 'non-exerciser' and a convenient time for the phone interview.

### Semi-structured interview guide

A semi-structured interview guide was developed for in-depth exploration based on core topical areas of the survey questionnaire. The guide was pilot-tested with 2 post-injury exercisers and 2 post-injury non-exercisers.

Questions focused on:

• Experiences with exercise before injury

• Experiences with exercise after injury

• Logistics of current exercise regimen

• Barriers and facilitators of exercise

• Perceived benefits of exercise

• Perceived impact of exercise on secondary conditions

• Experiences with pain management

• Future plans for exercise

We adopted a pragmatic participant-centered operationalization of 'exercise'. The determination of exercise status was based on self-reported information in the survey. 'Exercisers' were identified as those who reported being engaged in exercise activities either at home, in a gym or in both places. 'Non-exercisers' were identified as neither exercising at home or in a gym.

Consequently, in this paper, we will use the term 'exercise' to reflect both formalized exercise activities as well as physical activity initiated and carried out by individuals without the explicit intent to exercise (e.g. yard work, wheelchair pushing, etc.). Individuals, however, must have identified the activity as 'exercise'. This 'naturalistic' understanding of exercise reflects the recommendation by the Centers for Disease Control and Prevention and the American College of Sports Medicine that both lower-level intensity activity, as well as vigorous exercise, is beneficial in reducing the risk of heart disease and enhancing general fitness levels[35].

Similarly, our definition of 'secondary conditions' was participant-centred. The scope of conditions was defined by study participants.

### Procedure

All participants consented to the interviews before completing the first wave of the national survey. The study protocol, survey document, interview guide, informed consent and HIPAA documentation forms were approved by the MedStar Research Institute IRB in Hyattsville, MD. Essential demographic information was obtained at the time of the survey.

Each interview was conducted by telephone at a time convenient to participants. Interviews, conducted by an experienced interviewer, lasted between 20 and 30 minutes and were recorded with participant consent. Audio recordings were transcribed, and subsequently verified by two moderators.

### Data analysis

#### Quantitative

We conducted bi-variate, nonparametric analyses for differences in the demographic and clinical profiles between exercisers and non-exercisers. We computed Mann-Whitney U tests for continuous data (e.g. age, duration of injury), and χ^2 ^tests for independent samples for categorical data. The Fisher's Exact Test was used when cell sizes were smaller than 5.

#### Qualitative

We used an ethnographic approach and used a descriptive framework provided by the interview questions[36] to guide initial coding and content analysis. All transcripts were read by two analysts (authors of this manuscript) independently and initial thematic categories were recorded. Constant comparative coding[37] using TAMS Analyser for Mac OS X generated a total set of 55 codes, grouped as exercise type, barriers, facilitators, perceived benefits and secondary conditions. Thematic codes were discussed, negotiated and continuously refined between the two authors.

## Results

### Participant characteristics

Table 1 contains background characteristics of the 26 study participants.

**Table 1:** Participant characteristics (n = 26).

**Variable**	**'Exercisers'** N = 15	**'Non-exercisers'** N = 11	P ** p < .05
Age: Median (min, max)	52 (23, 74)	46 (34, 54)	.171
Sex:			.228
Female	4 (26.7%)	6 (54.5%)	
Male	11 (73.3%)	5 (45.5%)	
Ethnicity			.238
Caucasian/White	12 (80%)	11 (100%)	
Non-Caucasian	3 (20%)	0	
Education			.999
≤ 12 years	3 (20%)	3 (27.3%)	
> 12 years	12 (80%)	8 (72.7%)	
Income			.456
< $20	4 (28.6%)	3 (30%)	
$20 k – $60 k	3 (21.4%)	5 (50%)	
$61 k – $100 k	7 (50%)	1 (10%)	
> $100 k	0	1 (10%)	
Working			.781
Full/Part-time	6 (54.5%)	5 (45.5%)	
Not working	9 (60%)	6 (54%)	
Marital status			.462
Married/cohabitating	9 (60%)	5 (45.5%)	
Single/Living alone	6 (40%)	6 (54.5%)	
Duration of injury (Years): Median (min, max)	6 (1, 32)	20.5 (6, 32)	.029**
Injury Level			
C-level	9 (60%)	5 (45.5%)	.462
T-level	5 (33.3%)	4 (36.4%)	.999
Completeness of injury			
Complete	5 (33.3%)	6 (66.7%)	.206
Incomplete	10 (66.7%)	3 (33.7%)	.206
Wheelchair type			.729
Power chair	8 (53%)	5 (45%)	
Manual chair	6 (40%)	5 (45%)	
Does not use any chair	1 (7%)	1 (10%)	.819

There were no statistically significant differences between exercisers and non-exercisers with regard to age, gender, race, education level, employment status, marital status, injury level or completeness of injury. The only variable both groups differed on was 'duration of injury', with non-exercisers having a significantly longer median duration of injury. Despite the failed statistical significance between the groups in terms of reported household income, seven exercise-active participants indicated a household income above $60,000, while only two did so in the non-exercise group. The uneven group size may have masked group differences.

#### Pre-injury exercise experiences

Regardless of post-injury exercise status, most participants reported being exercise active pre-injury. Thus, not all pre-injury exercisers continued being exercise active after injury and several pre-injury non-active participants became active post-injury. Our finding suggests that pre-injury activity levels may prove to be a poor predictor of post-injury activity, a finding indicated elsewhere[38].

The conducted interviews revealed a wide range of factors that were identified as either facilitating or constraining of an individuals' level of activity. Each factor identified as a deterrent by one group, was often inversely identified as a facilitator by the other group, and vice versa. Understanding these psychosocial conditions and their elasticity can be essential to the success of promoting more active lifestyles post-injury. Often it is not one factor that facilitates or constrains, but a cluster of overlapping and intertwined factors, making unique each individuals experience while thus challenging our ability to identify consistent themes.

An important distinction among the facilitating and constraining factors reported by our participants, were whether their origin was socio-environmental or motivational. Participants identified motivation, or lack of motivation, as a major factor in the determination of their physical activity level. We present the varied roots of participants' motivation, or potential motivation. Secondly, participants identified socio-environmental barriers to or facilitators of an exercise active lifestyle. We present these exogenous factors and how they both helped or hindered participants' level of physical activity.

### Motivational triggers/constraints

Both exercisers and non-exercisers identified 'motivation' as the most critical factor to being and staying exercise active. Just as exercisers cited motivation as a strong facilitator of their being active, non-exercisers regarded their lack of motivation as a major constraint to an exercise-active lifestyle. Exercisers identified a variety of 'triggers' for their motivation while some non-exercisers suggested potential sources of motivation.

#### Perceived return on investment

One of the most commonly reported barriers to exercising among non-exercisers was the perceived 'limited return on investment' from aerobic exercise. The amount of time and energy needed to reach perceived beneficial levels of activity were identified as too demanding or unrealistic and failed to motivate some individuals.

'Yeah, it's just too much work for too little benefit. I've tried to do a few things here and there but, it just takes too much time and too much effort and I don't think the benefits out weight the costs.' ~Non-exerciser, male, 57, injury level unknown

'I find it, the exercise that I do, to be so different from the exercise I did before my injury in so many ways that it's also kind of psychologically difficult to exercise. It doesn't feel good... at all... I don't get sweaty... I don't get my heart rate up... I can feel my legs but not that well. So, it either makes me spasm or it like... or it just... wears me out.' ~Exerciser, male, 35, C4 incomplete

#### Adaptation and Outcome Expectancies

Some indicated that the activities they perceived themselves as capable of participating in, particularly modified sports such as tennis and basketball, were not comparable to the able-bodied version, allowing for frustration, disappointment and a disinterest in further pursuit.

'I couldn't do what I used to do. And there were a few things that I tried to do after the accident that were so discouraging that I quit. The main thing there was that I tried to play tennis in a wheelchair and I hated it. It wasn't the same game. I let it go because I couldn't stand facing it that way. I got on a horse eleven months after the accident, still in a wheelchair and it felt so good, since I had been on horses since I was four. And I think, I was reserving judgment because I didn't want to be disappointed the way I was with tennis.' ~Exerciser, female, 63, C5 incomplete

Several exercising participants, however, acknowledged that it took time to re-adjust expectations and exercise regimens post-injury. The rehabilitation process played a critical role in re-orienting many exercising participants towards new forms of exercise activities. It provided the motivational as well as the practical impetus to identify, shape and adopt acceptable and feasible exercise regimens that matched the individuals' lifestyle post-injury.

While there was confirmation of the importance of exercise, there was also acknowledgement that 'the way exercise happened' had to change.

*'Well it *[the injury]*changed how I exercised. I have to do everything sitting or laying down. It didn't change the fact that I knew that I had to keep my body fit and as young as I could.' ~Exerciser, male, 52, L5 incomplete*

#### Mental health and well-being

Several participants pointed to the perceived impact of exercise on their psychological well-being and mental health. Exercise was perceived as a means to prevent or reduce depressed mood, manage stress, control pain and provide structure and discipline. Respondents attributed enhanced quality of life to participation in exercise.

'Mentally I feel better that I'm accomplishing something and not just lying around. That I'm doing something good for myself.' ~Exerciser, male, 50, C7 incomplete

'The exercising definitely helps control the pain'. ~Exerciser, male, 52, L5 incomplete

'It just felt good, it made my body feel alive.' ~Exerciser, female, 63, C5 incomplete

Optimism or positive outlook on life appeared to be a facilitator of exercise activity. Exercise active participants made more statements that can be characterized as 'optimistic' and as relating to a positive outlook on life. Collectively, they were much more likely to make pointed, forward looking statements.

*'I think as I get older it *[exercise] *will lesson the complications I'll have in relation to my spinal cord injury. Less blood clogs, less muscle loss and flexibility. I don't want to worry about it later.' ~Exerciser, male, 35, T7 incomplete*

Although some non-exercisers did make statements that can be characterized as 'positive' and 'optimistic', collectively their statements were far less frequent, less specific and would be better described as 'hopeful'.

Another motivational factor was to stay as independent as possible and to reduce reliance on personal assistance.

'It took me a while to get into it. I was PO'd about the whole thing. But I knew I had to do it. If not I would be laying around and having someone push me and do things for me. And I wanted to live by myself. I didn't want a caregiver.' ~Exerciser, male, 50, C7 incomplete

#### Anticipated impact on health

Exercisers and non-exercisers clearly associated various health benefits to being physically active. While non-exercisers mostly perceived benefits in terms of general health and well-being and in the prevention of secondary conditions, especially pressure ulcers, exercisers detailed a broader range of benefits that they attributed directly to the exercise they were undertaking. Identification of specific exercise outcomes were a strong motivating force for continued exercise activity.

'*There is one exercise in particular with the weights that I've noticed...its the one where you sit and your knees are slightly bent and your feet are on a longish paddle and what you're doing is flexing your ankles up and down pushing the weight. Well I put low weights and I do one foot at a time and I'll do that to a count of 60 with each foot and after that... when I walk, those nerves that lift my foot up feel more normal and are working. They've been stimulated and it's easier to walk. It doesn't last forever, it goes away but I notice it after the exercise.' ~Exerciser, female, 63, C5 incomplete*

'*And at home also I mop the floor. Well I don't mop them, I bucket mop them. And I do that because I have to move my legs and I try to get my legs exercise. And I know I'm getting it because I get some feeling... and I can feel the muscles move...' ~Exerciser, male, 54, T12 incomplete*

Other benefits were seen with regard to general cardiovascular and respiratory fitness. Being physically active was also seen as improving motor control and maintaining physical strength.

'*I can tell a difference, if I haven't done much or have been kind of lazy for a day or two I can tell a difference. I'll feel more stiff. The more active I am, the more I move, the more I can do and the easier it is for me.' ~Non-exerciser, female, 48, T4 complete*

Several exercising participants reported being active to stave off the onset of secondary conditions, particularly pressure ulcers, urinary tract infections, bowel obstruction, bone loss and muscular atrophy.

*'You know, I think it has a lot to do with the bowel and bladder. When I don't exercise, I become congested and I'm just out of it. ... so I think all of that *[exercising] *helps me to be well all around, but most of all with my bladder and bowel changes'. ~Exerciser, male, 54, T12 incomplete*

While several exercisers used their fear of adverse health conditions as a motivator to exercise, some non-exercisers were constrained by a fear of causing 'damage' to oneself as a result of exercise.

'The other reason I don't walk more then I do is because I get nervous that with my abnormal gate I might wear out my joints also. I often wonder if because of the abnormal way I'm moving I'm putting abnormal stress on my joints. I think that if I were to go faster or longer I might get into trouble a little bit. I don't even know if I should be thinking about that, but I am.' ~Exerciser, male, 35, C4 incomplete

*'I still do it *[exercise]. *Partially out of fear. I don't want to get any worse. I'm a young guy. I'm only 34 and hopefully I have a lot of years ahead of me and I'd like to... you know, with spinal cord injury and aging there's a lot of complications and I want to prolong that as much as possible.' ~Exerciser, male, 35, C4 incomplete*

Exercisers reported that they were motivated to be exercise-active to maintain their health and well-being. They were concerned about secondary conditions and weight gain as a consequence of inactivity.

'Mostly just to keep up my strength and also being in my chair I put on quite a bit of weight and as a 24 year old girl that kind of bothers me. It's hard because you're not up walking around and stuff....' ~Exerciser, female, 24, C6 complete

For non-exercisers, health-related reasons, such as cardiovascular problems would provide an impetus for becoming more physically active.

'Well, if it were a matter of health. If you had to lose some weight or your blood pressure was too high. That kind of thing maybe. ...if I gain too much weight then it might be a motivational factor. I'd have to do something to burn some calories'. ~Non-exerciser, male, 50, C4

Several participants stressed the importance of physical activity for weight management.

'*I wasn't getting the results I wanted I guess. I had lost weight at some point and felt good about it, but gained it back and felt really bad. I was disappointed and wanted faster results to bring me back.' ~Exerciser, male, 35, T7 incomplete*

Several participants identified their excellent physical constitution before their injury as a major factor for good recovery. The value of maintaining health, strength, and fitness was perceived as an incentive for considering exercise post-injury.

*'... being in great shape before injury is a lifesaver. ...They were saying due to the physical shape I was in... that was one of the main factors' ~Non-exerciser, male, 41, C3 complete*.

### Socio-environmental resources/barriers

All participants, regardless of exercise status and motivation level recognized the impact and influence of social and environmental factors on their level of physical activity. Non-exercisers cited various barriers that prevented even the most motivated among them from becoming exercise active. While exercisers were often able to identify socio-environmental facilitators, many did so with a caveat, suggesting needs for improvement or acknowledging some level of constraint.

#### Information access

A lack of knowledge and resources were a commonly cited obstacle towards being active. Some non-exercisers expressed interest in being more physically active but were unsure of where to look for tips or assistance. For those living in a rural community with no local rehabilitation facility, this may be particularly true.

'I have always been trying to find a really good exercise routine with light weights for someone in a chair. They come out with exercise videos but they don't have the balance issues that people in chairs have to deal with. The bulkiness of the wheelchair, those kinds of things... and so I've never seen that. For me, that would be awesome. Just to have some kind of exercise video with suggested cardio and strengthening activities. I can't find it, whether it's out there, I don't know.' ~Non-exerciser, female, 45, T7 complete

Some emphasized that even their health care provider seemed unable to supply this information.

*'Yes, I would love to *[be exercise active] *and I want to but I think the idea is that a lot of the doctors who I go to, who are wonderful doctors, seem to be very knowledgeable just can't quite find a way to point me in the right direction of what to do and how to do it, or where to go. So I feel like my resources are a barrier to my future... With my latest doctor, for the past two years, in regards to bladder related, he would say 'you might feel better if you would just lose weight'. And I'm like, 'great, just tell me how I can do it.' And they're like... 'well...umm... I don't know where to send you. Have you looked online?' And so then I do the big online search and, like I said, there are exercise routines out there for people who are seated but they are not specific to wheelchair sitting. And nobody in the health care world have been able to provide me with any resources.' ~Non-exerciser, female, 45, T7 complete*

#### Accessibility

Of those non-exercisers indicating a willingness to exercise, one identified constraint was lack of accessibility, citing a void of accessible facilities, or having to travel long distances to reach such facilities. Some exercisers however specifically cited accessible facilities as an important facilitator for their active lifestyle post-injury.

'They have automatic doors and a family locker room that I use because they have a roll in shower I can transfer over to. Private lockers and such.' ~Exerciser, male, 50, C7 incomplete

Several non-exercisers indicated that direct access to equipment would increase their likelihood of being active. As with the general population, time and a busy lifestyle can always make healthy living seem difficult to attain, and thus assigning time to be physically active becomes an inconvenience.

*'I would say, mostly because of the activities of my daily life. I'm married. I have children. I work. And so, it's just daily activities like everyone else would probably say. I'm very busy and that's *[exercise] *the least important aspect... it shouldn't be, but it is.' ~Non-exerciser, female, 45, T7 complete*

#### Support and Personal Assistance

A lack of personal assistance was noted as a significant barrier by participants in both subsets.

'I could probably get on a cycle machine that is motorized to move my legs, but... I guess the main obstacle is having someone else take up their time to help me do it. I would need at least one or two people.' ~Non-exerciser, male, 50, C4

Even motivated exercisers with access to equipment must cope with this particular constraint, as this participant details.

*'Yeah, I definitely do *[want to continue being active]. *It's just difficult to find someone to help is the main problem. It's not necessarily my motivation, or not having the equipment, its just getting the help to help me do it. I enjoy getting in my stander, it's just a pain because I can't get in it by myself.' ~Exerciser, female, 25, C6 complete*

Some exercisers relied heavily on the use of personal assistance.

'I have a personal care assistant and he spends six hours a day with me and part of that of course is bathing and so forth, but four hours of that is exercising. I'm a tetraplegic. I can walk a little distance. We do balance exercises; work my arms and hands. And at night, in my garage I have a hand cycle, and I use it.' ~Exerciser, male, 70, C3 incomplete

Several non-exercisers viewed 'social support' as a factor that could potentially allow them to become more exercise active. A few exercisers also acknowledged that the 'fun' element of being active with others was something they enjoyed and looked forward to.

'I think I would probably need to be in something more structured where I would have to go and do it with someone else. To really get myself to do it at this age...' ~Non-exerciser, female, 44, C5 incomplete

#### Affordability and Insurance Coverage

In addition to costs associated with purchasing home equipment, participants, regardless of exercise status, noted insurance coverage as a barrier to being active; particularly true for those using Medicare or Medicaid. Concerns most emphasized were those of coverage limitations.

'There is one other thing I'd like to mention to you and that's insurance. It doesn't cover a lot of equipment that would be useful, like a stand. The ability to stand will decrease your probability of getting pressure sores. Gravity alone is going to help alone with UTIs and things like that... Insurance will normally not cover equipment to stand. ...Braces so I can stand... insurance doesn't pay for braces' ~Non-exerciser, female, 48, T4 complete

*'If I have neck pain they *[Medicare] *might give me a week or two of therapy but that's all. And I believe I would have been a lot further ahead as far as mobility if I were allowed to have more therapy.' ~Exerciser, female, 66, T7 incomplete*

## Discussion

Our findings suggest that participants' physical activity levels are contingent on a combination of motivational and socio-environmental factors, varying from case to case, and making generic exercise prescription problematic. Reported barriers and facilitators to exercise may be differentiated by these motivational or socio-environmental origins. All participants identified multiple factors as having influence over their level of physical activity. In sorting the common themes of our interviews, we recognize and emphasize the interwoven relationship between motivational triggers/constraints and socio-environmental resources/barriers. An attempt to simplify or minimize the potential inter-dependence of these factors would be to distort the experiences reported. In fact, what we may best discern from these shared experiences is that no one factor acts as a function of an individuals' level of physical activity; it is instead a cluster of varying factors, with shifting degrees of severity and influence from case to case. This paper seeks only to heighten and highlight individual factors within this more complex context.

The Theory of Planned Behavior[39] posits that physically active behavior is a function of intention, which is influenced by attitudes, subjective norms and perceived behavioral control. Other studies have successfully applied this theory to the SCI population[27,28] and our findings further validate its usefulness. Exercisers' attitudes were linked to the knowledge of the benefits of being physically active while non-exercisers considered the level of physical investment required to reach the perceived degree of benefit as unrealistic, unattainable or simply too demanding. Exercisers and non-exercisers reported similar levels of pre-injury physical activity, suggesting a shared norm of exercise as an essential part of life, yet the motivation needed to comply with this standard was a point of divergence between the two groups post-injury. Finally, non-exercisers reported frustration with physical limitations and unhappiness with existing exercise options, while exercisers identified physical therapy and training received in rehabilitation as major facilitators. A positive experience with exercise options during rehabilitation may shape the perception of behavioral control in terms of one's ability to be exercise active post-injury and lay the foundation for exercise engagement. Participants in this study, and elsewhere[25,30] identified their time in rehabilitation and physical therapy as critical for their current level of exercise commitment, while several non-exercisers noted a lack of support or recommendation to exercise from their physician, a problem recorded elsewhere[26,32]. It is also worth noting that non-exercisers had a significantly longer median duration of injury, compared to exercisers. The possibility that duration of injury has a potentially negative effect on the intentions to exercise should be explored further.

Study participants saw multiple benefits of regular exercise in terms of maintaining physical health and preventing secondary conditions. Apart from general cardiovascular fitness, several participants were concerned about pressure ulcers, urinary tract infections and respiratory problems. Some perceived the threat of these conditions as a motivator for maintaining their exercise regimen. The risk of weight gain was also a contributing factor for several participants. The psychological benefits that many respondents derived from their exercise engagement are consistent with findings from the literature [40-43]. While these health related factors were reported as facilitators in this study, they have been reported as benefits of exercise elsewhere[23,31]. The difference between a facilitator and a benefit is important to note. While perceived health benefits may act as a facilitator of *continued *exercise, it would seem that only the anticipation of such benefits would facilitate initial engagement.

In theoretical terms it may be argued that people who participate in regular exercise show greater 'self-determination'. Self-determination theory[44,45] posits that individuals need to develop a sense of autonomy and competence, which is essential for a process of internalization and integration of health behaviors (i.e. exercise). Autonomy implies that individuals value and prioritize behaviors and make them integral to their life-style. Additionally, people may feel more inclined to be physically active if they perceive themselves as confident and competent. People who experience a greater degree of autonomy may also be more likely to learn new behaviors and feel competent. In our study several exercisers acknowledged that their exercise routines and strategies had changed post injury but that the reasons for being physically active had not. It may be argued that they managed to integrate physical activity effectively in their post-injury life and felt competent in exploring alternative means of achieving the expected exercise benefits. Other participants who had been physically active before their injury however, did not perceive these benefits (e.g. aerobic gains) and did not make exercise an integral part of their lives post-injury. There is some indication that individuals with long-standing exercise routines may see greater personal benefits than external incentives (e.g. weight loss; appearance) to pursue regular physical activities[46]. Another element of the self-determination theory is 'relatedness', referring to the quality of the patient-practitioner relationship and how it may shape individuals' motivation to engage in behavior change. As noted, several respondents in this study felt that health professionals did not provide sufficient support and recommendations for exercise post-injury. Self-determination theory however, has not been studied in people with SCI. Similarly, a better understanding of psychological constructs, such as 'optimism'[47], with regard to influencing outcome expectancies related to exercise may further complement the picture.

The identification of socio-environmental barriers, especially those focused on accessibility and disability-specific knowledge of health providers, are not unique to the problem of exercise, and have been reported in conjunction with access to health services[26,32,48-51]. A greater proportion of participants expressed interest in being physically active than actually were, a finding reflected elsewhere[24,26]. The removal of socio-environmental barriers could prove to be one of the most effective facilitators, thus allowing a motivated individual access to choosing an exercise active lifestyle.

It is important to emphasize that the socio-environmental barriers identified by non-exercisers were often, in their inverse form, factors that facilitated a physically active lifestyle for exercisers. Motivation aside, exercisers were able to be active because of the availability of an accessible community-based facility, or because of their capability to maintain home equipment. Non-exercisers often regarded the lack of such facilities and/or equipment as a barrier, a finding reflected in prior studies[26,52]. The role of personal assistance is equally noteworthy as many exercisers, particularly those relying on home equipment were able to do so only with the assistance of another. The absence of such help was identified as a major constraint by participants in both groups.

Even those for whom cost was not a barrier acknowledged its pervasive role in determining access. Facilities, personal assistance and home equipment are often available with a financial cost; one that several exercisers admitted at being able to meet. For those relying on insurance coverage, particularly Medicaid and Medicare, limitations of coverage emerged as an often impenetrable barrier, potentially denying individuals access to continuous and long-term physical activity.

More comprehensive and multi-level efforts are needed to address the physical health promotion needs of individuals with spinal cord injuries and other physical disabilities as Healthy People 2010 goals are far from being met.

### Limitations

The study has several limitations. Its qualitative nature does not necessarily allow findings to be generalized to a larger population. The selection of subgroup participants (exercise vs. non-exercise) was based on a pool of people with SCI who had been previously included in a non-random sample. One of the principal limitations of both the larger survey study, from which the sub-sample was drawn, and the resulting subsets was the limited representation of ethnic minority groups. Even though there were no statistically significant differences between exercisers and non-exercisers in our selected subgroup, more individuals in the exercise group had higher disposable incomes and reported having more personal exercise equipment and assistance. This may be a finding in itself. Economic inequities among people with SCI may drive differential access to exercise information and support.

## Conclusion

Most people with SCI are principally motivated to engage in exercise so to maintain health and prevent secondary conditions. Labeling individuals simply as 'non-compliant' without a full appreciation of their motivational constraints and socio-environmental barriers to exercise is not helpful. Successful behavior modification requires the consideration of both these types of factors. While improvements in providing better access to affordable facilities and personal assistance, especially in rural communities, is essential in developing accessible, inclusive, and equitable exercise support and health promotion programs, equal consideration must be given to the unique socio-environmental realities faced by individuals if such programs are to reach their effective potential. These broadened considerations allow clinicians and public health professionals to arrive at an understanding of physical activity that is not solely focused on restoring, improving or maintaining function, but enhancing general health and wellbeing. Closing education gaps and addressing professional 'blind-spots' among health care professionals may additionally help abate some of the obstacles that currently deny motivated individuals with SCI participation in healthy living activities.

## Competing interests

The authors declare that they have no competing interests.

## Authors' contributions

TK was the principle investigator of this study and involved in the original protocol design. MK and TK developed the semi-structured interview guide together. All interviews were conducted by MK. Interview transcriptions, subsequent analysis and development of this manuscript were performed by both MK and TK. Both authors approved this final manuscript.

## Pre-publication history

The pre-publication history for this paper can be accessed here:


